# Association of autistic personality traits with the EEG scores in non-clinical subjects during the facial video viewing

**DOI:** 10.18699/vjgb-24-108

**Published:** 2024-12

**Authors:** A.N. Savostyanov, D.A. Kuleshov, D.I. Klemeshova, M.S. Vlasov, A.E. Saprygin

**Affiliations:** Institute of Cytology and Genetics of the Siberian Branch of the Russian Academy of Sciences, Novosibirsk, Russia Scientific Research Institute of Neurosciences and Medicine, Novosibirsk, Russia Novosibirsk State University, Novosibirsk, Russia; Institute of Cytology and Genetics of the Siberian Branch of the Russian Academy of Sciences, Novosibirsk, Russia Trofimuk Institute of Petroleum Geology and Geophysics of the Siberian Branch of the Russian Academy of Sciences, Novosibirsk, Russia Siberian State University of Telecommunications and Informatics, Novosibirsk, Russia; Institute of Cytology and Genetics of the Siberian Branch of the Russian Academy of Sciences, Novosibirsk, Russia Scientific Research Institute of Neurosciences and Medicine, Novosibirsk, Russia; Altai State Pedagogical University, Biysk Branch named after V.M. Shukshin, Biysk, Russia; Institute of Cytology and Genetics of the Siberian Branch of the Russian Academy of Sciences, Novosibirsk, Russia Scientific Research Institute of Neurosciences and Medicine, Novosibirsk, Russia

**Keywords:** information-digital platforms in medicine ., neurocomputation technologies, resting-state EEG, autistic personality traits, Broad Autism Phenotype, self-referential processing, default-mode network, информационно-цифровые платформы в медицине, нейровычислительные технологии, ЭЭГ покоя, аутистические черты, расширенный аутистический фенотип, самореференция, дефолт-система мозга

## Abstract

A software information module of the experimental computer platform “EEG_Self-Construct” was developed and tested in the framework of this study. This module can be applied for identification of neurophysiological markers of self-referential processes based on the joint use of EEG and facial video recording to induce the brain’s functional states associated with participants’ personality traits. This module was tested on a group of non-clinical participants with varying degrees of severity of autistic personality traits (APT) according to the Broad Autism Phenotype Questionnaire. The degree of individual severity of APT is a quantitative characteristic of difficulties that a person has when communicating with other people. Each person has some individual degree of severity of such traits. Patients with autism are found to have high rates of autistic traits. However, some individuals with high rates of autistic traits are not accompanied by clinical symptoms. Our module allows inducing the brain’s functional states, in which the EEG indicators of people with different levels of APT significantly differ. In addition, the module includes a set of software tools for recording and analyzing brain activity indices. We have found that relationships between brain activity and the individual level of severity of APT in non-clinical subjects can be identified in resting-state conditions following recognition of self-referential information, while recognition of socially neutral information does not induce processes associated with APT. It has been shown that people with high scores of APT have increased spectral density in the delta and theta ranges of rhythms in the frontal cortical areas of both hemispheres compared to people with lower scores of APT. This could hypothetically be interpreted as an index of reduced brain activity associated with recognition of self-referential information in people with higher scores of autistic traits. The software module we are developing can be integrated with modules that allow identifying molecular genetic markers of personality traits, including traits that determine the predisposition to mental pathologies.

## Abstract (ru)

В рамках проводимого исследования разработан и апробирован программно-информационный модуль экспериментально-компьютерной платформы “EEG_Self-Construct”, позволяющий выявлять нейрофизиологические маркеры самореферентных процессов на основе совместного использования ЭЭГ и регистрации видеозаписей лица для индукции функциональных состояний головного мозга, ассоциированных с личностными особенностями участников. Этот модуль был апробирован на группе неклинических участников с разной степенью выраженности аутистических личностных черт (АЛЧ), измеренных с помощью опросника расширенного фенотипа аутизма. Степень индивидуальной выраженности АЛЧ – это количественный показатель, который характеризует затруднения, возникающие у человека при коммуникации с другими людьми. У каждого человека имеется некоторая индивидуальная степень выраженности таких черт. Высокие значения аутистических черт определяются у пациентов с аутизмом. Однако существуют также люди, у которых высокие значения АЛЧ не сопровождаются клинической симптоматикой. Разработанный нами модуль дает возможность индуцировать функциональные состояния головного мозга, в которых ЭЭГ-показатели людей с разным уровнем АЛЧ достоверно различаются. Кроме того, модуль включает комплект программного обеспечения для регистрации и анализа индексов мозговой активности. Нами установлено, что зависимости между мозговой активностью и индивидуальным уровнем выраженности АЛЧ у неклинических испытуемых могут быть выявлены в условиях функционального покоя, следующих за распознаванием самоотнесенной информации, тогда как распознавание социально нейтральной информации не индуцирует процессы, связанные с аутистичностью. Показано, что у людей с высокими значениями АЛЧ наблюдаются повышенные показатели спектральной плотности в диапазонах дельта- и тета-ритмов в лобных отделах обоих полушарий в сравнении с людьми с низкой степенью аутистичности. Это может быть гипотетически интерпретировано как индекс сниженной мозговой активности, ассоциированной с распознаванием самоотнесенной информации у людей с высокой аутистичностью. Разрабатываемый нами программный модуль может быть интегрирован с модулями, позволяющими выявлять молекулярно-генетические маркеры личностных черт, включая черты, определяющие предрасположенность к психиатрическим патологиям.

## Introduction

The development of new approaches to identifying predisposition to certain types of behavior, including an increased risk of developing mental disorders, is based on testing individuals using genetic, neurophysiological and behavioral methods, accumulating experimental information in databases and analyzing it using a wide range of information technologies (Ivanov et al., 2022; Lin et al., 2022).

According to modern concepts, autism is a disease that is associated with disturbances in the brain and manifests itself in the social sphere (Baron-Cohen, 2002; Lavenne-Collot et al., 2023). This disease manifests itself in three domains of behavior: social interaction, communication (use of verbal and non-verbal stimuli), as well as limited and repetitive patterns in behavior, interests and activities (Baron-Cohen, 2009; Murray et al., 2017). In the 1980s, autism was recognized as a spectrum of conditions (disorders), which can be individual for each patient (Lovaas, 1987).

There is no strict boundary between a “healthy person” and an “autistic person”, since each person can be assigned a certain rate of some autistic personality traits (APT) measured by the Broad Autism Phenotype Questionnaire, BAPQ (Piven et al., 1997). The higher the rate of APT, the more the subject’s behavior resembles that of an autistic person. It is believed that the manifestation of APT is clinical in nature if its rate exceeds a certain threshold. However, there is a phenomenon of “non-clinical autism”, when a person with an expressed APT does not consider it necessary to seek medical help. At the same time, a significant part of such “non-clinical autistic persons” turn out to be adapted people who, during their lives, demonstrate a level of social success that is no different from individuals with low rates of autistic traits. It is assumed that there are some compensatory mechanisms that may be formed depending on the influence of the environment and can both weaken and strengthen the manifestation of APT in subjects (Frith, 1991; Georgiades et al., 2017).

Since autism and APT are associated with behavioral difficulties in social communication, most neurophysiological (Tsai et al., 2013; Tseng et al., 2015) and genetic (Genovese, Butler, 2023) studies compare the brain responses of individuals with different degrees of autistic traits to the presentation of external stimuli, the recognition of which is essential for the regulation of interpersonal communication. For experimental research of the phenomenon of autism, approaches such as psychological testing using questionnaires, recording and analysis of EEG under stimulation conditions are used. Facial photographs (Harms et al., 2010; Tseng et al., 2015) or speech tasks (Tsai et al., 2013) are usually used as stimuli. However, some studies demonstrate the association of the severity of autism with brain activity under restingstate conditions without recognition of external stimuli (Harikumar et al., 2021).

An effective method is the registration of a facial video to induce psychological states that differ in participants with different degrees of expression of personality traits (Si et al., 2024).

Another approach used is to record the EEG without any additional stimulation. It is based on the hypothesis about the functional role of the default mode network of the brain in organizing self-reference processes. The default mode network is a set of cortical areas that demonstrate increased activation under resting-state conditions, but decrease the level of activation when performing tasks associated with attention to external stimulation. The default mode network is considered as a brain structure involved in the assessment of socially significant stimuli that the subject attributes to oneself (Northoff et al., 2005). It is assumed that clinical forms of autism are accompanied by a decrease in the activity of the default mode network (Ronde et al., 2024). The functioning of the default mode network can be associated not only with the characteristics of individuals’ social behavior, but also with the characteristics of their genome (Fanelli et al., 2024).

Previously, we proposed an approach for joint registration and processing of EEG and facial video that allows combining brain activity analysis with assessment of facial muscle dynamics (Savostyanov et al., 2022). In this study, we propose a methodology based on the use of video fragments obtained at the first stage of the study to stimulate participants at later stages of the study. As shown below, this approach provides useful information for identifying markers of autistic traits in non-clinical subjects.

To provide information support for the conducted research, we are developing the “EEG_AutisticTrait” software information module, which is an important component of the “EEG_Self-Construct” experimental computer module. It provides a full cycle of information support for research, including: (a) accumulation and storage of the results of examining people using psychological, neurophysiological and genetic methods that make it possible to identify individual characteristics of social communication associated with autism; (b) computer processing of experimental data using regression, correlation and factor analysis methods that compare behavioral and neurophysiological indicators (Si et al., 2024); (c) visualization of primary experimental data and results of data analysis.

The fundamental novelty of the proposed approach is that time intervals of EEG recordings under resting-state conditions in the intervals between recognition of selfreferential or non-self-referential stimuli are used to identify neurophysiological markers of APT. This approach allows inducing mental states associated with self-reference in the intervals of functional rest.

## Materials and methods

The sequence of stages of the experimental computer module “EEG_Self-Construct” and the list of software tools required for the implementation of these stages are presented in Table 1. The module contains both software products developed by ICG SB RAS staff and programs taken from open sources. All modules allow for a full cycle of data collection and processing required to establish markers of autistic personality traits.

**Table 1.**
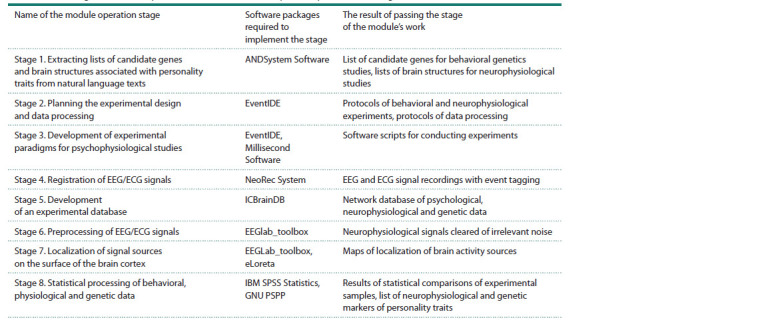
List of stages of module operation and software tools required to perform each stage

Study participants. The study involved volunteers, among which students of Novosibirsk State University prevailed. The sample included 43 participants aged from 18 to 48 years (19 males and 24 females). All participants had no neurological or mental disorders at the time of the study and did not use any psychoactive substances or pharmacological drugs. Participants gave informed consent to undergo the experimental study in accordance with the Helsinki Declaration on Biomedical Ethics. The experimental protocol was approved by the Ethics Committee of the Research Institute of Neuroscience and Medicine.

Psychological testing was performed using a special Internet form implemented on the Yandex platform by ICG SB RAS staff. All participants filled out the Russian-language version of the BAPQ to assess the severity of autistic traits according to the Broad Autism Phenotype Questionnaire (Hurley et al., 2007, translated by M.S. Vlasov). This test includes 36 questions concerning a person’s ability to control one’s behavior in social situations. In addition, the participants filled out psychological questionnaires on personal and situational anxiety by C. Spielberger (Spielberger, 1970; Russian adaptation by (Khanin, 1976)), a questionnaire for assessing personality traits by L. Goldberg “Markers of the Big Five Factors” (translated and validated by G.G. Knyazev et al. (2010), a questionnaire on affiliation with one’s family (Cross et al., 2000), and a questionnaire on emotional intelligence (Knyazev et al., 2012).

Experiment. The program for conducting the experiment is implemented on the Inquisit platform (https://www. millisecond.com/). In the experiment, the participants fulfilled three conditions. In the first condition, the EEG was recorded for 12 minutes without a functional load. The subject had three 2-minute intervals with closed eyes and three 2-minute intervals with open eyes. During the intervals when the subject opened one’s eyes, a black computer screen was presented to the subject. During this period, the subject’s face was recorded along with the EEG for all 12 minutes. The second and third conditions differed from the first in that in the second condition, with open eyes, the subject watched a video recording of his or her own face, obtained from the first condition, and in the third condition, he/she was shown a video recording of a stranger’s face (always a man for a male subject, and a woman for a female subject). The order of the second and third tasks was changed randomly.

EEG registration and processing. The NeoRec software (by “Medical Computer Systems”, https://mks.ru/) was used to register neurophysiological data. EEG was registered using a 130-channel amplifier NVX-132, Russia, 128 EEG channels located according to the international 5-5 % scheme with a reference electrode Cz, ground electrode AFz, bandwidth 0.1–100 Hz, signal sampling frequency 1000 Hz. In addition to EEG, EOG and ECG were additionally registered.

Muscle and other artifacts were removed from the EEG using independent component analysis with the EEGlab_toolbox software package (Delorme, Makeig, 2004; https://sccn.ucsd.edu/eeglab/index.php). Then, fragments corresponding to periods when the participant sat with eyes closed were extracted from the EEG recordings. Further analysis was performed only for those intervals of the EEG recordings in which the participant did not see either video recordings or a blank screen, but which were recorded immediately after observing the corresponding stimuli. After extracting these EEG fragments, they were divided into two-second time intervals. Further analysis was performed using the eLoreta software package (Pascual-Margui, 2002; https://www.uzh.ch/keyinst/loreta.htm).

In our case, the neurophysiological states detected using eLoreta were compared with the psychological characteristics of the subjects to determine the markers of APT. For each two-second interval, the spectral density values were calculated in the frequency of delta (2–4 Hz), theta (4–8 Hz), alpha-1 (8–10 Hz), alpha-2 (10–12 Hz), beta-1 (12–16 Hz), beta-2 (16–20 Hz), beta-3 (20–25 Hz) and gamma (25–35 Hz) bands. Then, for each participant, the total spectrum indicator was calculated for the entire EEG testing interval separately for each of the three experimental conditions (for each participant, from 150 to 170 two-second intervals were used for this). The spectra were calculated independently for each of the 128 EEG channels included in data processing. A 3000 ms EEG recording segment with a sampling frequency of 1000 Hz after the onset of the block was used to calculate the spectral density of the sources in eLoreta (Pascual-Margui, 2002).

Statistical analysis. The validity of psychological tests was assessed using the IBM SPSS software package, IBM, https://www.ibm.com/spss. Regression analysis was performed in the eLoreta package to find the dependence of spectral density on the indicators of individual BAPQ score independently for each of the three experimental conditions. Additional correction for multiple comparisons was not performed.

## Results

Results of psychological testing

To assess the reliability of the Russian version of the BAPQ test, we determined the internal consistency of responses to 36 items of this questionnaire using Cronbach’s alpha. The Cronbach’s alpha value was 0.838, which indicates a fairly high internal consistency. In addition, we assessed the correlation of individual BAPQ scores with scores on various scales of well-validated psychological measures. Table 2 shows the correlation between autistic traits (BAPQ scores) and other personality traits assessed in this study. The BAPQ score correlates reliably positively with anxiety and negatively with extroversion, the ability to express positive emotions and affiliation with the family.

**Table 2.**
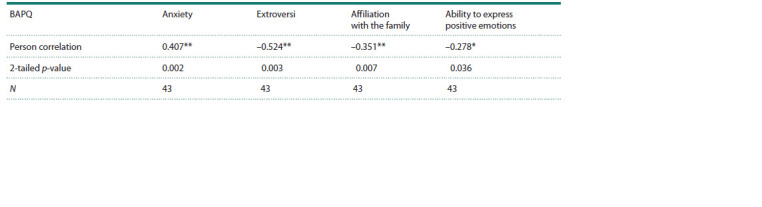
Correlation between autistic traits (BAPQ score) and other personality traits * Significant correlation, p-value <0.05 (two-tailed). ** Significant correlation, p-value <0.01 (two-tailed).

eLoreta results for detecting effects of autistic traits

Correlations between BAPQ autistic traits scores were statistically significant only for the “own face” condition ( p = 0.0340) in the delta (2–4 Hz) and theta (4–8 Hz) bands (see the Figure). For both bands, eLoreta revealed a positive association between the spectral density scores and individual severity of autistic traits in the frontal cortex of both hemispheres, i. e. higher BAPQ autistic traits scores corresponded to higher spectral density scores. There was no significance for the “blank screen” condition ( p = 0.28640). For the “another person’s face” condition ( p = 0.0932), the p-value was close to, but did not reach, significance

**Fig. 1.**
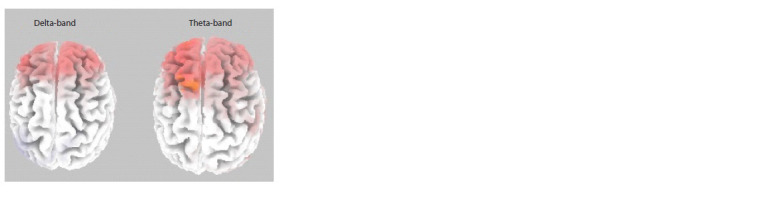
Correlation of the spectral density in the delta (2–4 Hz) and theta (4–8 Hz) bands with the severity of autistic traits (measured by BAPQ) in a group of 43 participants for EEG intervals with eyes closed between viewing one’s own face The cortical areas showing positive correlations of autistic traits with spectral density (p <0.04) are marked in red. A significantly positive association is observed between autistic traits and spectral density in the frontal areas of both hemispheres.

## Discussion

Identification of neurophysiological markers of personality traits, including traits associated with predisposition to diseases, involves the use of complex multicomponent tools for planning experiments, collecting, storing and analyzing data, comparing the results of different studies and organizing access to different programs and the data obtained with their help. An important component of such tools is the opportunity to develop and implement new paradigms for conducting neurophysiological research. For example, in (Si et al., 2024), a software module was developed to identify cross-national characteristics in the processes of self-attribution of information to the subject oneself or to other people, which is crucial for the search for markers of depression.

In the search for markers of predisposition to psychiatric disorders, an important task is the reconstruction and analysis of gene networks underlying the regulation of psycho-emotional states in humans and animals (Savostyanov, Makarova, 2024). An example of a module aimed at reconstructing and comparing gene networks of anxiety in mice and humans is described in (Savostyanov, Makarova, 2024). Using this module, it is possible to identify brain structures in which differential gene expression is detected in animals that differ in their level of anxiety. In the future, such structures can be considered as areas of interest for identifying neurophysiological markers of anxiety disorder in humans.

The software-information module “EEG_AutisticTrait” was tested to identify neurophysiological markers of autistic personality traits. Using a special Yandex platform, comprehensive testing of participants was conducted using several questionnaires, including a test for individual expression of autistic personality traits (the Russian version of BAPQ). The Cronbach’s alpha for the Russian version of BAPQ was 0.83, which indicates a fairly high internal consistency of this questionnaire. Negative correlations of autistic personality traits with extroversion, emotional intelligence and affiliation with the family, and positive correlations between autistic personality traits and anxiety were also found, which is in good agreement with the general understanding of psychologists about autistic traits.

At the neurophysiological level, positive correlations were found between BAPQ scores and the spectral density in the delta and theta bands for the experimental condition associated with self-referential visual information, but no reliable relationships were found for the conditions following viewing a socially neutral stimulus (blank screen) or information related to other individuals. According to the literature (Knyazev, 2007), high values of the spectral density of the delta and theta rhythm under resting-state conditions are most often interpreted as an indicator of reduced functional brain activity. With this approach, our results can be hypothetically explained as a correlate of reduced brain activity in conditions following the presentation of self-referential information in individuals with more vivid autistic traits compared to individuals with lower levels of autistic traits.

Significantly, we identified neurophysiological correlates of autistic traits only for the self-referential condition. In the socially neutral condition, there was no tendency for BAPQ scores to be related to brain activity, whereas for the “another person’s face” condition, there was a marginal statistical tendency for the result to be significant. It can be assumed that resting-state EEG activity in non-clinical subjects is weakly associated with their level of autism, which explains the failure of previous attempts to identify any relationships between autistic traits and resting-state EEG in such participants. However, viewing video recordings related to the participant oneself (and to a lesser extent, to other people) activates processes in the brain associated with the recognition of socially significant information, which makes EEG indices more dependent on autistic traits than in the case of viewing socially neutral stimuli.

## Conclusion

The approach we propose is based on the integration of psychological and neurophysiological methods of data collection and analysis. In the future, it is planned to evaluate the dependence of autistic traits on the genetic characteristics of the subjects. It is also desirable to evaluate the effect of the expression level of various genes in the brain on the severity of personality traits. The assessment of the level of gene expression in the brain cannot be performed on a living person, which suggests the need to combine data obtained on people and on experimental animals (Savostyanov, Makarova, 2024). Such a study requires the development of special tools for the accumulation, storage and analysis of data, which will be created on the basis of the Bioinformatics and Systems Computational Biology platform. In the future, this tool can be used to assess the neurophysiological correlates of various personality traits in healthy controls and subjects with different pathologies, which will make it possible to conduct new comprehensive studies within the framework of system neurobiology.

## Conflict of interest

The authors declare no conflict of interest.
